# Meningitis due to *Fusobacterium necrophorum *in an adult

**DOI:** 10.1186/1471-2334-4-24

**Published:** 2004-08-05

**Authors:** SreeNeelima Garimella, Aparna Inaparthy, Thomas Herchline

**Affiliations:** 1 Department of Internal Medicine, Wright State University School of Medicine, Dayton, Ohio 45409, USA; 2 Division of Infectious Diseases, Department of Internal Medicine, Wright State University School of Medicine, Dayton, Ohio 45409, USA

## Abstract

**Background:**

*Fusobacterium necrophorum *may cause a number of clinical syndromes, collectively known as necrobacillosis. Meningitis is a significant cause of mortality, rarely reported in the adult population.

**Case presentation:**

We report a fatal case of meningitis, caused by *Fusobacterium necrophorum*, secondary to otitis media in an alcoholic male. Diagnosis was delayed due to the typical slow growth of the organism. The clinical course was complicated by encephalitis and by hydrocephalus. The patient failed to respond to metronidazole and penicillin. The patient died on day 12 from increased intracranial pressure and brain stem infarction.

**Conclusions:**

This case emphasizes the need for a high index of clinical suspicion to make the diagnosis of *Fusobacterium necrophorum *meningitis. We recommend the use of appropriate anaerobic culture techniques and antimicrobial coverage for anaerobic organisms when the gram stain shows gram negative bacilli.

## Background

*Fusobacterium necrophorum *may cause a number of clinical syndromes, collectively known as necrobacillosis. Meningitis is a significant cause of mortality, rarely reported in the adult population. Diagnosis is often delayed by difficulties encountered in isolating the organism. Here, we report a fatal case of meningitis from complicated otitis media caused by this organism.

## Case presentation

A 51 year male was brought to the emergency department (ED) by his family for confusion and shaking episodes. The patient was very lethargic in the ED and was intubated for airway protection. Family reported that he had not been well for several months, but the family was not able to define any specific symptoms until the past few days when he reported right ear pain. The patient had been given a prescription for erythromycin within the previous week for a diagnosis of otitis media; the patient had not taken any doses for at least two days. The only significant past medical history was of ongoing alcohol abuse without intravenous drug use. The family reported that he had no alcohol intake over the two days prior to presentation. Vitals on presentation: temperature 98.5°F (36.9°C), heart rate 125/min, respirations 25/min, blood pressure 219/121, Oxygen saturation 96 % on room air. On physical exam he was lethargic, the right tympanic membrane was erythematous with decreased movement on pneumatic otoscopy. The neurological exam revealed no focal deficits.

**Figure 1:**
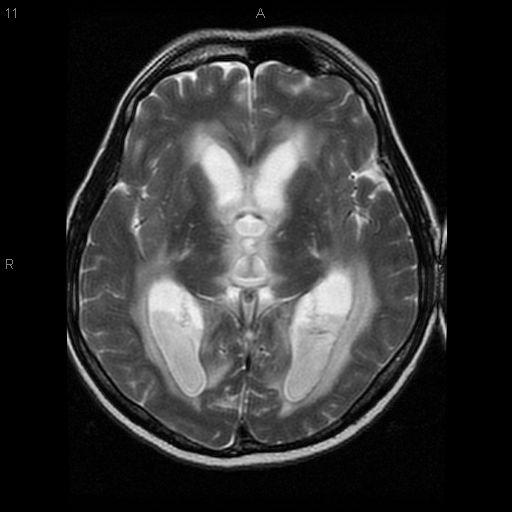
Magnetic resonance imaging of brain on hospital day six. T2 weighted image showing obstructive hydrocephalus and ventriculitis.

**Figure 2:**
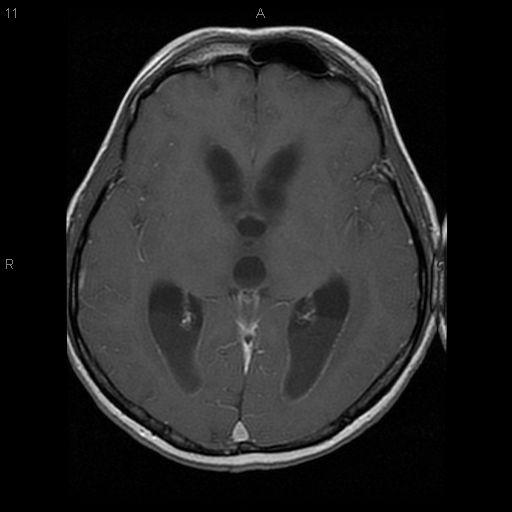
Magnetic resonance imaging of brain on hospital day six. T1 weighted, post-gadolinium image showing obstructive hydrocephalus and ventriculitis.

**Figure 3:**
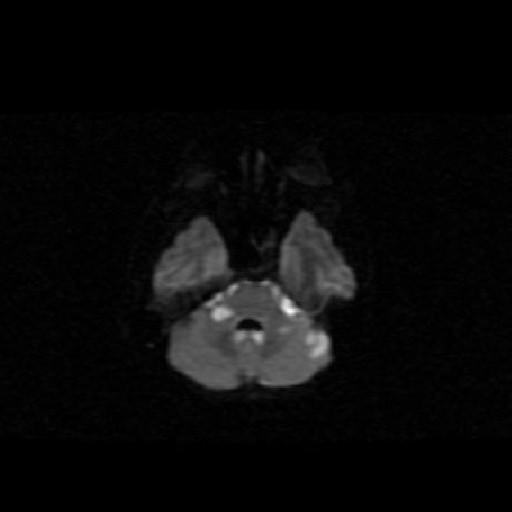
Magnetic resonance imaging of brain on hospital day six. Diffusion image showing acute infarcts in the cerebellum.

**Figure 4:**
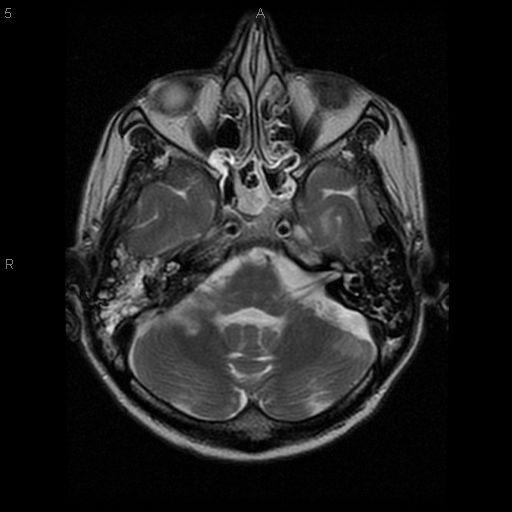
Magnetic resonance imaging of brain on hospital day six. T2 weighted image showing fluid within the right mastoid air cells.

A non-contrasted head CT scan showed no abnormalities; there was no evidence of an intracranial bleed or elevated intracranial pressure. Lumbar puncture revealed markedly purulent CSF with 338,400 white cells per cubic millimeter (94% neutrophils), glucose was less than 2 mg/dl and protein was 3544 mg/dl. Gram stain showed numerous gram negative bacilli. He was initially treated with high dose dexamethasone, along with ceftriaxone, ampicillin and vancomycin. When gram negative bacilli were identified in the CSF, the antibiotic regimen was revised to ceftazidime, levofloxacin and metronidazole. There was a decline in mental status despite aggressive antibiotic therapy. He continued to require complete ventilatory and pressor support to maintain adequate blood pressure and oxygenation. Physical examination revealed up-going plantar reflexes on day 6. MRI brain showed extensive infarction of the brain stem, obstructive hydrocephalus, severe basilar meningitis, and ventriculitis (see figures 1,2,3,4). A ventricular drain was placed in an attempt to decrease intracranial pressure. Repeat CSF analysis on day 9 showed 1800 white cells per cubic millimeter and protein was 118 mg/dl. At this time, initial culture of the CSF was reported to be growing *F. necrophorum. *Penicillin G was added with discontinuation of ceftazidime and levofloxacin. The patient further deteriorated and the neurological exam revealed absent corneal reflexes, and no response to painful stimulus. EEG was done to assess brain function, which revealed evidence of brain death. The caloric test also confirmed brain death.

## Discussion

*Fusobacterium *is an anaerobic, non spore forming gram negative rod which belongs to the family of *Bacteroidaceae*. It is a part of normal flora which is found in mouth, upper respiratory tract, gastrointestinal tract and vagina. It can cause local infections like pharyngitis, tonsillitis, mastoiditis or can cause severe bacteremic illness like meningitis. Other central nervous system complications caused by *Fusobacterium *include cranial nerve palsy, sinus venous thrombosis, and brain abscess [1,2]. In one recent series of brain abscesses, *F. necrophorum *was the most common anaerobe isolated, found in 33% of patients [2]. The term "necrobacillosis" or "Lemierre syndrome" is used for the severe bacteremic illness caused by *F. necrophorum*. Lemierre syndrome has been described to progress through three stages [3]. The primary infection is pharyngitis in the majority of patients. The second stage is invasion into the pharyngeal space with the development of internal jugular septic thrombophlebitis. The third stage is metastatic spread of the infection.

In one series of *F. necrophorum *meningitis, middle ear infection was the source for 75% of the cases [4]. Other predisposing infections include sinusitis, pharyngitis and lung infections [5]. Diagnosis is often delayed by the difficulties in isolating and identifying the organism. A high index of suspicion is necessary in the diagnosis of this infection. There have been over 20 reported cases of meningitis due to *Fusobacterium *[1,4,6-19], only one of which was in an adult [6]. Despite appropriate antibiotic therapy, the outcome is poor with the mortality rate from meningitis due to Fusobacterium as high as 33% with residual sequelae common among survivors (60%) [7,8]. Although the antibiotic regimen of choice has not been established metronidazole seems to be a useful agent. Some authors have suggested the addition of penicillin G to treat this infection [8]. It has been recommended that metronidazole be administered for at least 6 weeks [6]. Relapse is possible if the treatment is discontinued prematurely [4,6].

## Conclusions

This case shows the severity of illness that can result from infection with *F. necrophorum*. Anaerobic organisms should be considered as potential causative agents of meningitis when routine cultures are negative. Routine cultures of cerebrospinal fluid do not include the use of anaerobic growth media. Therefore, appropriate anaerobic culture techniques should be employed when sinus, otitic or mastoid symptoms precede or accompany the onset of meningitis in children or adults. The presence of irregularly stained gram negative rods in the CSF or meningitis unresponsive to empiric antibiotics should also raise the suspicion of anaerobic infection. The addition of metronidazole should be considered in these cases.

## Competing interests

None declared.

## Authors' contributions

AI cared for the patient. SG drafted the manuscript. TH cared for the patient. All authors reviewed and approved the final manuscript.

## Pre-publication history

The pre-publication history for this paper can be accessed here:
